# Effects of Hygiene and Defecation Behavior on Helminths and Intestinal Protozoa Infections in Taabo, Côte d’Ivoire

**DOI:** 10.1371/journal.pone.0065722

**Published:** 2013-06-20

**Authors:** Thomas Schmidlin, Eveline Hürlimann, Kigbafori D. Silué, Richard B. Yapi, Clarisse Houngbedji, Bernadette A. Kouadio, Cinthia A. Acka-Douabélé, Dongo Kouassi, Mamadou Ouattara, Fabien Zouzou, Bassirou Bonfoh, Eliézer K. N’Goran, Jürg Utzinger, Giovanna Raso

**Affiliations:** 1 Department of Epidemiology and Public Health, Swiss Tropical and Public Health Institute, Basel, Switzerland; 2 University of Basel, Basel, Switzerland; 3 Département Environnement et Santé, Centre Suisse de Recherches Scientifiques en Côte d’Ivoire, Abidjan, Côte d’Ivoire; 4 Unité de Formation et de Recherche Biosciences, Université Félix Houphouët-Boigny, Abidjan, Côte d’Ivoire; 5 Unité de Formation et de Recherche Sciences Naturelles, Université Nangui Abrogoua, Abidjan, Côte d’Ivoire; 6 Unité de Formation et de Recherche Communication, Milieu et Société, Université Alassane Ouattara, Bouaké, Côte d’Ivoire; 7 Unité de Formation et de Recherche Sciences de la Terre et des Ressources Minières, Université Félix Houphouët-Boigny, Abidjan, Côte d’Ivoire; The George Washington University Medical Center, United States of America

## Abstract

**Background:**

More than 1 billion people are currently infected with soil-transmitted helminths and schistosomes. The global strategy to control helminthiases is the regular administration of anthelmintic drugs to at-risk populations. However, rapid re-infection occurs in areas where hygiene, access to clean water, and sanitation are inadequate.

**Methodology:**

In July 2011, inhabitants from two villages and seven hamlets of the Taabo health demographic surveillance system in south-central Côte d’Ivoire provided stool and urine samples. Kato-Katz and ether-concentration methods were used for the diagnosis of *Schistosoma mansoni*, soil-transmitted helminths (*Ascaris lumbricoides*, *Trichuris trichiura*, and hookworm), and intestinal protozoa. Urine samples were subjected to a filtration method for the diagnosis of *Schistosoma haematobium*. A questionnaire was administered to households to obtain information on knowledge, attitude, practice, and beliefs in relation to hygiene, sanitation, and defecation behavior. Logistic regression models were employed to assess for associations between questionnaire data and parasitic infections.

**Principal Findings:**

A total of 1,894 participants had complete data records. Parasitological examinations revealed prevalences of hookworm, *S. haematobium*, *T. trichiura*, *S. mansoni*, and *A. lumbricoides* of 33.5%, 7.0%, 1.6%, 1.3% and 0.8%, respectively. *Giardia intestinalis* and *Entamoeba histolytica*/*E. dispar* were detected in 15.0% and 14.4% of the participants, respectively. Only one out of five households reported the presence of a latrine, and hence, open defecation was common. Logistic regression analysis revealed that age, sex, socioeconomic status, hygiene, and defecation behavior are determinants for helminths and intestinal protozoa infections.

**Conclusions/Significance:**

We found that inadequate sanitation and hygiene behavior are associated with soil-transmitted helminths and intestinal protozoa infections in the Taabo area of south-central Côte d’Ivoire. Our data will serve as a benchmark to monitor the effect of community-led total sanitation and hygiene education to reduce the transmission of helminthiases and intestinal protozoa infections.

## Introduction

Hundreds of millions of people are still affected by neglected tropical diseases (NTDs), particularly in the developing world due to parasitic worm infections (helminthiases) [1], [2]. Taken together, soil-transmitted helminthiasis and schistosomiasis are responsible for 8.5 million disability-adjusted life years (DALYs) with more than 1 billion people infected [3]–[5]. Diseases caused by intestinal protozoa infections, such as giardiasis and amebiasis also cause considerable morbidity and mortality [6]–[9].

Current helminthiases control programs focus on preventive chemotherapy, that is the regular administration of anthelmintic drugs to at-risk populations, particularly school-aged children [10], [11]. However, preventive chemotherapy does not prevent re-infection, which might occur rapidly [12], [13]. Additionally, there is considerable concern about the development of drug resistance in the era of preventive chemotherapy, as experience has shown in livestock [14]. Although, the importance of integrated control approaches for the interruption of transmission of helminthiases is well established since almost a century [15], [16], current control efforts emphasize drug interventions, and do not give sufficient attention to hygiene behavior, clean water, and adequate sanitation [17]–[19]. Indeed, data from 2010 suggest that 2.6 billion people lacked access to some kind of improved sanitation [20]. To contribute to the achievement of several of the millennium development goals (MDGs), ongoing efforts to control NTDs have to be maintained and further intensified, including complementary approaches for prevention and control [21].

In July 2011, a project emphasizing an integrated control approach targeting intestinal parasites was launched in the Taabo health demographic surveillance system (HDSS) in south-central Côte d’Ivoire. The main objective is to assess the impact of community-led total sanitation (CLTS) and health education on the incidence of helminths and intestinal protozoa infections, implemented alongside preventive chemotherapy. CLTS not only focuses on the construction of latrines, but also on local knowledge, attitude, practice, and beliefs (KAPB) related to hygiene and defecation behavior, which play a key role for sustainability [22]. Through a participatory grassroots approach, CLTS aims to achieve and sustain an open defecation-free status of communities [23]. To our knowledge, the effect of CLTS on re-infection patterns with helminths and intestinal protozoa infections has yet to be determined. Here, we present helminth and intestinal protozoa infection profiles in a selection of villages and hamlets of the Taabo HDSS, including associations between infection and people’s KAPB related to hygiene and defecation behavior during the baseline cross-sectional survey. Our data will serve as a benchmark for monitoring the longer term impact of CLTS on people’s health and wellbeing.

## Methods

### Ethics Statement

This study received clearance from the ethics committees of Basel (Ethikkommission beider Basel; reference no. 177/11) and Côte d’Ivoire (Comité National de l’Ethique et de la Recherche; reference no. 13324 MSLS/CNER-P). Study participants were informed about the aims, procedures, and potential risks and benefits. Participants and parents/guardians of minors provided written informed consent (signature of a witness for illiterate participants). Participation was voluntary and people could withdraw from the study at any time without further obligation. To guarantee anonymity, each study participant was given a unique identification number.

At the end of the parasitological survey, anthelmintic treatment was administered to all people in the study villages and hamlets regardless of infection status and participation (single 400 mg oral dose of albendazole for individuals aged ≥2 years) [10], [11]. Additionally, participants aged ≥4 years who were diagnosed for *Schistosoma* spp. were given a single oral dose of praziquantel (40 mg/kg, using a “dose pole”) [10], [11]. Individuals who required other specific medical interventions were referred to the next health care facility. No treatments were given to participants identified with intestinal protozoa infections, as the results from the sodium acetate-acetic acid-formalin (SAF)-fixed stool samples subjected to an ether-concentration method were only available several weeks after completion of the field work and intestinal protozoa infection are often self-limiting.

### Study Area and Population

The study was conducted in the Taabo HDSS, located in a primarily rural part of south-central Côte d’Ivoire [24]–[26]. General living standards are low. For instance, 71% of households lacked a toilet facility and two-third of the households used unprotected surface water (e.g., rivers and lakes) as drinking water according to the latest available data from the Taabo HDSS. Soil-transmitted helminthiasis, schistosomiasis, onchocerciasis, and lymphatic filariasis control activities have been implemented within the Taabo HDSS (preventive chemotherapy, using albendazole, praziquantel, and ivermectin) since 2008 and are on-going with yearly drug interventions. At the time of the execution of the current study, preventive chemotherapy was the main strategy implemented in the study area.

**Figure 1:**
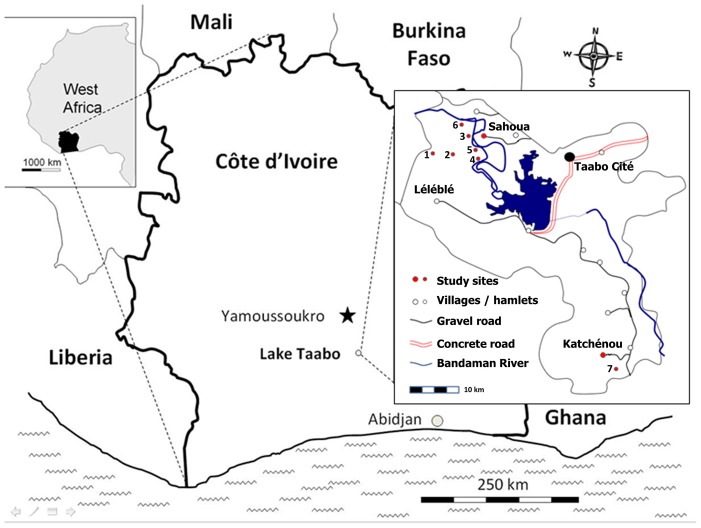
Map of the study area in Taabo, situated in south-central Côte d’Ivoire. The study was carried out in two villages (Sahoua, Katchénou) and seven hamlets (1 = Beh N’Guessankro, 2 = Allah Thérèsekro, 3 = Yobouékro, 4 = Ouattafouékro, 5 = Kouadio Kouamékro, 6 = Boussoukro, 7 = Amani Kouadiokro) that are part of the Taabo health demographic surveillance system. Results presented here pertain to the baseline cross-sectional parasitological and questionnaire surveys conducted in July 2011.

The study presented here was implemented in two villages (i.e., Katchénou and Sahoua), and seven hamlets of different villages, Yobouékro (belonging to Sahoua), Ouattafouékro and Kouadio Kouamékro (Ahondo), Boussoukro (Tokohiri), Amani Kouadiokro (Sokrogbo), and Beh N’Guessankro and Allah Thérèsekro (Léléblé) (Figure 1). These villages and hamlets were purposely selected because of their small population sizes, and the relatively homogeneous population structure. All inhabitants of the villages and hamlets were targeted as study population, using the readily available Taabo HDSS database.

### Study Design and Procedures

In July 2011, just shortly before the annual round of preventive chemotherapy, a cross-sectional survey was carried out to assess the baseline parasitological and KAPB situation. This cross-sectional survey was part of a larger, still ongoing project aiming to assess the effect of CLTS on helminths and intestinal protozoa re-infection patterns. This larger project consists of a baseline cross-sectional survey (design, field and laboratory procedures, questionnaire survey, and results are presented in this paper), implementation of CLTS combined with health education, and a 1-year follow-up survey.

Before commencement of the study, villages/hamlets were visited by the research team to get approval from the local authorities and to announce the exact date of the sampling day. The day before the sampling, participants were given empty plastic containers for stool and urine collection. Participants were invited to bring early morning stool and urine samples to a central place in the village/hamlet. For parasitological examinations, fecal and urine samples were transferred to our mobile field laboratories in Léléblé and Sokrogbo, or the laboratory of the hospital in Taabo-Cité.

Duplicate Kato-Katz thick smears were prepared on microscope slides from each stool sample, using standard templates holding 41.7 mg of feces. Slides were examined under a microscope and the eggs of *Schistosoma mansoni*, *Ascaris lumbricoides*, *Trichuris trichiura*, and hookworm were counted by experienced laboratory technicians the same day, and recorded for each species separately [27]. Ten milliliters of vigorously shaken urine were filtered, the filter placed on a microscope slide and a drop of Lugol’s iodine added. The slides were examined under a microscope and the number of *S. haematobium* eggs counted [28]. For quality control, all slides were read independently by different laboratory technicians. When inconsistencies were detected, the discordant slides were re-examined and the results discussed until agreement was reached.

Additionally, from each stool sample, 1–2 g was transferred into a small plastic tube filled with 10 ml of SAF [29]. The SAF-fixed stool samples were forwarded to a laboratory at the Centre Suisse de Recherches Scientifiques en Côte d’Ivoire (CSRS; Abidjan, Côte d’Ivoire) for further examination to detect intestinal protozoa infections. In brief, samples were processed using an ether-concentration method and the slides were analyzed by experienced laboratory technicians under a microscope [30]. Standard protocols were followed and intestinal protozoa (*Blastocystis hominis*, *Chilomastix mesnili*, *Entamoeba coli*, *Entamoeba hartmanni*, *Entamoeba histolytica/E. dispar*, *Endolimax nana*, *Giardia intestinalis*, and *Iodamoeba bütschlii*, recorded semi-quantitatively [31].

A questionnaire was administered to all households at the day of stool and urine sampling. The households were visited by a researcher accompanied by a trained field enumerator who speaks the local languages. Whenever the head of a household was present, he/she was interviewed; otherwise a present adult household member was interviewed. The questionnaire was designed in a structured manner with closed questions to obtain quantitative data for the analyses. The questionnaire consisted of basic questions on demographic factors (e.g., age, sex, ethnicity, and education), socioeconomic indicators (e.g., possession of a number of household assets), and KAPB. Topics covered by the KAPB were: (i) sanitation and defecation behavior (e.g., place of defecation, use of latrine); (ii) open defecation (e.g., reasons for open defecation, problems of open defecation); (iii) hygiene behavior (e.g., hand washing after defecation); (iv) opinions, taboos, and beliefs (e.g., preoccupations, gender-specific latrine use); and (v) intestinal parasitic infections (e.g., prevention, transmission, signs, and symptoms). The questionnaire was piloted in 10 households in a village not otherwise involved.

### Statistical Analysis

Data were double-entered and cross-checked in EpiInfo version 3.5.1 (Centers for Disease Control and Prevention; Atlanta, United States of America) and analyzed in STATA version 10.0 (Stata Corp.; College Station, United States of America). Only participants with complete datasets (i.e., those with duplicate Kato-Katz thick smears, one SAF-fixed fecal sample, and one urine filtration) were included in the final analyses. For each participant, the arithmetic mean egg count was calculated and used to stratify the infection intensities (mean number of eggs per gram of stool (EPG)) into light, moderate, and heavy infections using cut-offs commonly employed by the World Health Organization (WHO) [32]. Participants were stratified into five age groups (i.e., <5; 5–14; 15–24; 25–40; and >40 years). For the variables and summary statistics of the KAPB questionnaire, frequency tables with indicators of central value and dispersion were calculated. Furthermore, the several categories of the KAPB questionnaire were coded for their importance with a value of 0 if the category was not mentioned at all, a value of 1 after a probed answer, and a value of 2 after a spontaneous answer [33]. The KAPB questionnaire data gathered at the unit of the household served as individual values for every participant living in a specific household, which might slightly distort our results for logistic regression. Participants with a particular helminth or intestinal protozoan infection were compared to participants not infected with that species. Test statistics included chi-square (χ^2^), Fisher’s exact test, Wilcoxon rank-sum, Kruskal-Wallis, two sample *t*-tests, and logistic regression models adjusted for participants’ socioeconomic status, age group, and sex. Hence, these characteristics were included wherever these parameters showed significant association with infection. Furthermore, all logistic regressions were corrected for potential clustering at the unit of the village/hamlet.

The socioeconomic status was calculated using a household asset-based approach [34]. Household asset weights were determined using principal component analysis (PCA). Missing values were replaced by the mean of the particular asset. Only binary variables were used for household assets. Household assets were excluded to make the first principal component (PC) stand for more than 30% of the variability. Greatest weight were given to the possession of a television (0.34), followed by the presence of a shower with cement floor (0.33), and the possession of a video recorder (0.33). The calculated scores were added up for each household and subsequently ranked according to the total score. The households were then separated into wealth quintiles: (i) poorest, (ii) very poor, (iii) poor, (iv) less poor, and (v) least poor. To estimate inequities in parasitic infection prevalence related to the participants’ socioeconomic status, the concentration index (CI) was used [35], that arises from the concentration curve. It quantifies the degree of socioeconomic-related inequality in a health variable and is twice the area between the concentration curve and the 45-degree line that is called the line of equality. The CI is 0 if there is no socioeconomic-related health variable. When the CI becomes negative then the curve lies above the line of equality indicating that there is a disproportionate concentration of the health variable among the poor and, *vice versa*, it takes a positive value if the concentration of the health variable is among the wealthier. Significance of the CI was assessed using standard deviations [36].

## Results

### Study Participation

**Figure 2:**
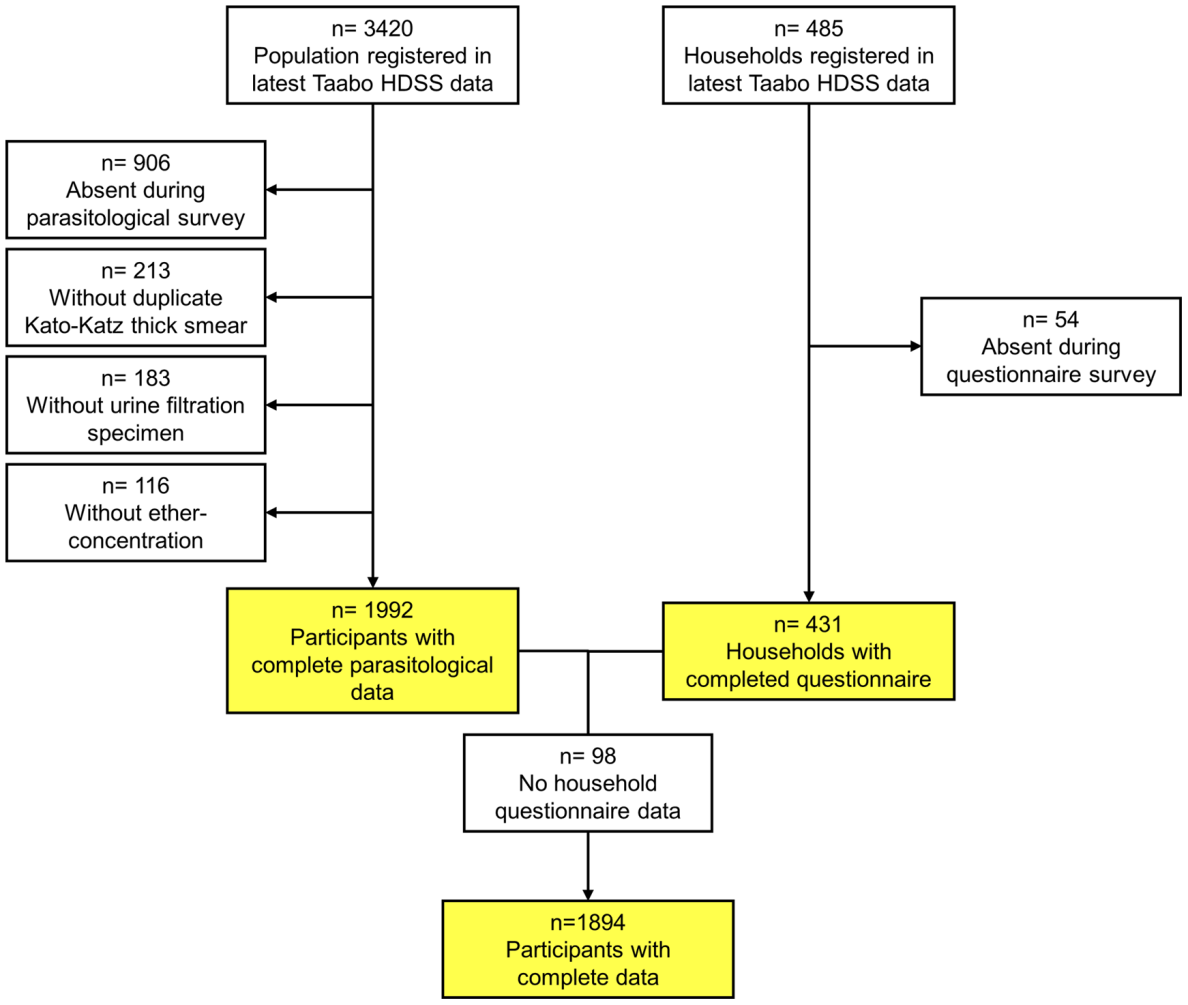
Flow chart showing the study cohort and compliance with emphasis on the three different samples considered in the analysis. The study was carried out in the Taabo health demographic surveillance system in south-central Côte d’Ivoire in July 2011. The three sub-samples pertain to participants with complete parasitological data, households with complete questionnaire data, and participants with complete parasitological data from a household with complete questionnaire data.

From 3,420 registered people in 485 households in the selected villages and hamlets in the Taabo HDSS, 2,514 individuals were present during the cross-sectional parasitological survey. As shown in Figure 2, 213 participants lacked duplicate Kato-Katz thick smears (no stool sample was provided), 183 had no urine filtration done (no urine sample was provided), and 116 had missing ether-concentration data (insufficient stool provided to perform the test). Complete parasitological data were available from 1,992 individuals (58.2% based on the registered population).

In 54 households, adult members were either absent or refused to participate in the questionnaire survey. Interviews were conducted in the remaining 431 households (88.9%). For regression analysis, 98 participants dropped, due to missing questionnaire data leading to a final study sample of 1,894 people (55.4% of the registered population).

### Parasitological Results

Among those 1,992 participants with complete parasitological data, we found prevalences for hookworm, *S. haematobium*, *T. trichiura*, *S. mansoni*, and *A. lumbricoides* of 33.5%, 7.0%, 1.6%, 1.3%, and 0.8%, respectively (Table 1). Only very few individuals were identified with moderate or heavy helminth infection intensities, with the exception of *S. haematobium* (25.9% of the infections were classified as heavy, i.e., ≥50 eggs/10 ml of urine). The prevalences of the pathogenic intestinal protozoa *G. intestinalis* and *E. histolytica*/*E. dispar* were 15.0% and 14.4%, respectively. The most common intestinal protozoa were *E. coli* and *B. hominis* with respective prevalences of 45.0% and 35.4%.

**Table 1:** Helminth infection prevalence and intensity among 1,992 participants in Taabo, south-central Côte d’Ivoire, in July 2011.

Parasite species	Infected (%)	Minimum egg count	Maximumegg count	Infection intensity^a^		
				Light	Moderate	Heavy
Hookworm	667 (33.5)	12	13,584	616 (96.7)	14 (2.2)	7 (1.1)
*T. trichiura*	32 (1.6)	12	2,316	25 (83.3)	5 (16.7)	0 (0.0)
*S. mansoni*	26 (1.3)	12	2,520	20 (87.0)	2 (8.7)	1 (4.3)
*A. lumbricoides*	15 (0.8)	24	5,832	8 (88.9)	1 (11.1)	0 (0.0)
*S. haematobium*	139 (7.0)	1	685	103 (74.1)	n.d.	36 (25.9)

Infection intensities (mean egg count) were split into light, moderate, and heavy infections using WHO guidelines [10], [11].

a Number of infected participants stratified by infection intensities (values in brackets as percentage, %).

n.d., not defined.

Males were significantly more likely to be infected with hookworm than females (38.8% *vs.* 28.2%; χ^2^ = 25.49, *p*<0.001). The same patterns were found for *E. coli* (50.6% *vs.* 39.4%; χ^2^ = 25.08, *p*<0.001) and *E. nana* (31.8% *vs.* 25.2%; χ^2^ = 10.69, *p* = 0.001). In contrast, females were more likely to be infected with *T. trichiura* compared to males (2.2% *vs.* 1.0%; χ^2^ = 4.62, *p* = 0.032).

**Figure 3:**
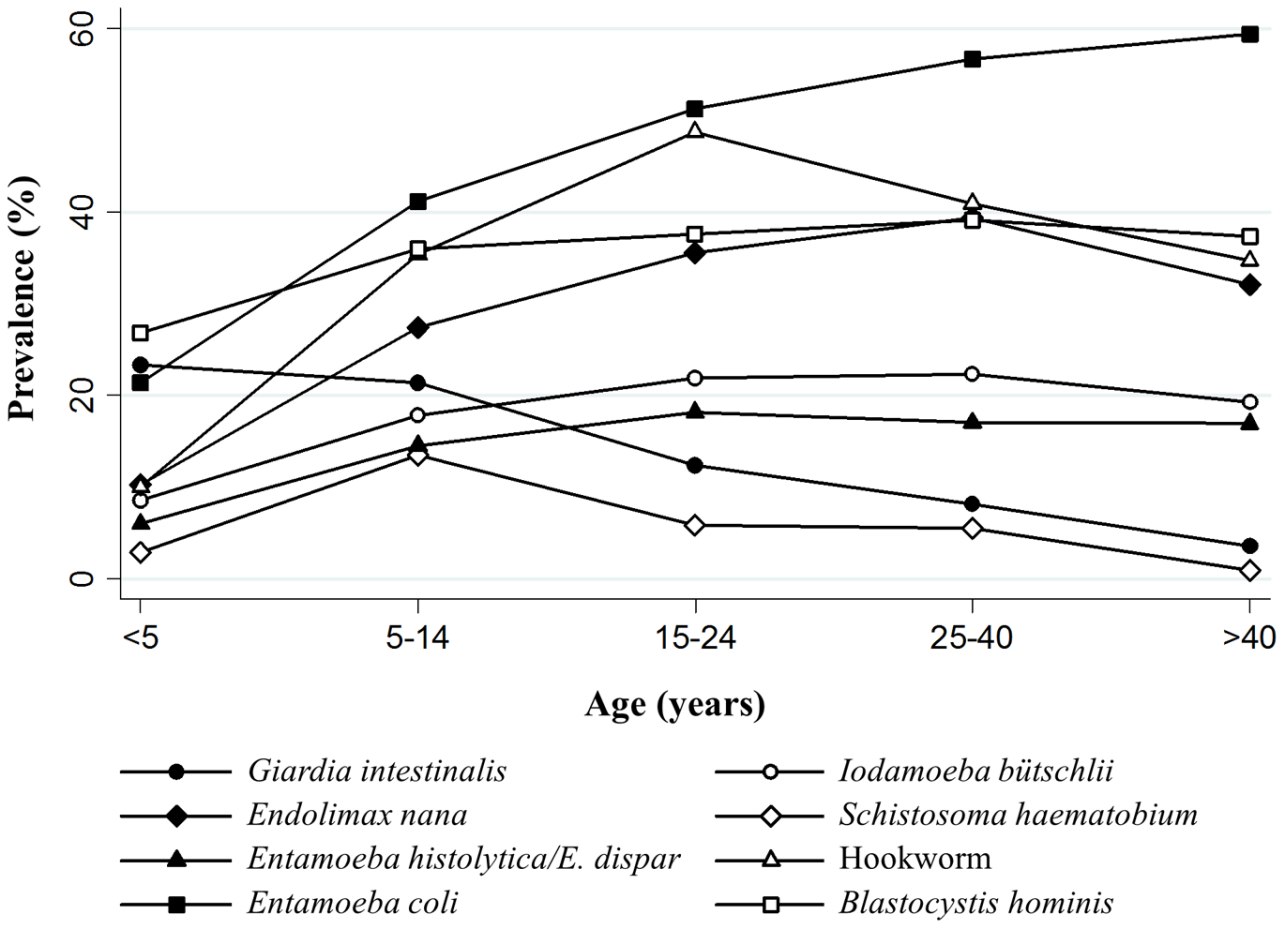
Age-prevalence curves of investigated parasites. The results of the intestinal protozoa and helminth infections arise from the baseline cross-sectional survey carried out in July 2011 among community members of two villages and seven hamlets in the Taabo health demographic and surveillance system, south-central Côte d’Ivoire. *Trichuris trichiura*, *Schistosoma mansoni* and *Ascaris lumbricoides* are not displayed due to very low prevalence.

Several intestinal parasites were significantly associated with age group, including hookworm (χ^2^ = 123.35, degree of freedom (d.f.) = 4, *p*<0.001), *S. mansoni* (χ^2^ = 14.11, d.f. = 4, *p* = 0.007), *S. haematobium* (χ^2^ = 74.68, d.f. = 4, *p*<0.001) and six of the eight encountered intestinal protozoa (*E. histolytica*/*E. dispar*, *E. coli*, *E. nana*, *I. bütschlii*, *G. intestinalis*, and *B. hominis*). Age-prevalence curves are shown in Figure 3. Participants of poorer households were significantly more often infected with hookworm (CI = −0.0266, standard error (SE) = 0.0085), *T. trichiura* (CI = −0.2774, SE = 0.1230), *E. histolytica/E. dispar* (CI = −0.1072, SE = 0.0242), *I. bütschlii* (CI = −0.0414, SE = 0.0189), and *G. intestinalis* (CI = −0.0548, SE = 0.0162). However, the prevalence of *S. haematobium* was significantly higher in the richer participants compared to their poorer counterparts (CI = 0.2249, SE = 0.0382).

### Results of the KAPB Survey

Table 2 shows the demographic and socioeconomic characteristics among the households, stratified by wealth quintiles. Muslims were more frequently part of the least poor quintile, compared to Christians and animists. Household size steadily increased from poorest to least poor. Household assets such as electricity, latrine, television, and a motorcycle were more often reported by the least poor quintile. The poorest more often obtained their drinking water from the nearby Bandama River or other unprotected open surface water bodies than their wealthier counterparts who were more likely to use a village pump as source of drinking water.

**Table 2:** Characteristics of the 431 households, participating in the knowledge, attitude, practice, and beliefs survey, stratified by wealth quintiles.

Characteristics	Total (*n = *431)	Wealth quintiles (%)					Ratio (poorest/least poor)
		Poorest(*n = *85)	Very poor (*n = *85)	Poor (*n = *81)	Less poor (*n = *91)	Least poor (*n = *89)	
**Sex (%)**							
Male	59.2	50.6	55.3	61.7	65.9	61.8	0.82
Female	40.8	49.4	44.7	38.3	34.1	38.2	1.29
**Age (years)**							
Mean (SD)	40.3 (14.2)	37.6 (12.3)	39.4 (13.4)	37.5 (11.8)	43.3 (16.1)	43.0 (15.8)	
Median (Q1–Q3)	39 (30–48)	36 (29–45)	38 (30–47)	37 (27–45)	41 (31–53)	40 (32–55)	
**Status of the respondent (%)**							
Household chief	57.3	55.3	56.5	59.3	60.4	55.1	1.00
Wife	28.5	37.7	31.8	24.7	26.4	22.5	1.68
Son or daughter	7.7	3.5	5.9	9.9	8.8	10.1	0.35
Other	4.4	3.5	4.7	2.5	4.4	7.9	0.44
Brother or sister	2.1	0.0	1.2	3.7	0.0	4.5	0.00
**Religion (%)**							
Christian	44.1	47.6	45.8	46.9	51.1	29.6	1.61
Muslim	23.0	17.4	12.2	15.3	17.4	37.8	0.46
Animist	29.1	29.8	34.9	32.1	26.7	22.7	1.31
Other religions	3.8	5.2	7.1	5.7	4.8	9.9	0.53
**Educational attainment (%)**							
No education	66.6	64.7	64.7	72.8	68.1	62.9	1.03
Primary school	18.3	25.9	22.4	17.3	13.2	13.5	1.92
Secondary school	9.7	7.1	9.4	8.6	11.0	12.4	0.57
Koranic school	4.4	2.4	3.5	1.2	5.5	9.0	0.27
University	0.9	0.0	0.0	0.0	2.2	2.2	0.00
**Reading-writing ability (%)**							
Reading	27.8	31.8	32.9	24.7	24.2	25.8	1.23
Writing	27.6	31.8	32.9	23.5	24.2	25.8	1.23
**Household size**							
Mean (SD)	7.3 (4.3)	5.5 (2.8)	6.5 (3.5)	6.7 (3.8)	8.5 (5.0)	9.2 (4.9)	0.60
Median (Q1–Q3)	6 (5–9)	5 (4–7)	6 (5–8)	6 (4–9)	8 (5–11)	8 (6–11)	0.75
**Household assets (%)**							
Shower	88.4	88.2	92.9	70.0	91.2	97.7	0.90
Bicycle	79.3	64.7	80.0	77.5	82.4	91.0	0.71
Radio	72.8	65.9	77.7	70.0	72.5	77.5	0.85
Motorcycle	22.3	4.7	17.7	20.0	24.2	43.8	0.11
Latrine	20.7	10.6	9.4	12.5	33.0	38.2	0.28
Television	18.4	1.2	1.2	3.8	17.6	65.2	0.02
Electricity	14.2	1.2	1.2	4.9	11.0	51.1	0.02
**Drinking water source (%), multiple answers possible**							
Pond/river	58.8	81.5	61.9	50.6	55.0	46.0	1.77
Pump	37.7	18.5	35.7	43.0	37.4	52.9	0.35
River	37.4	60.5	40.5	27.9	27.5	32.2	1.88
Pond	21.6	21.0	21.4	22.8	28.6	13.8	1.52
Cistern	19.4	16.1	17.9	17.7	30.8	13.8	1.17
Well	1.9	1.2	3.6	0.0	2.2	2.2	0.55

The study was carried out in the Taabo health demographic surveillance system in south-central Côte d’Ivoire in July 2011. Questionnaires were conducted with the household chief if present; otherwise the next higher household member was interviewed.

Q1–Q3 stands for first quartile to third quartile, defining the interquartile range.

Most interviewees (98.6%) said that they would wash their hands regularly. The most frequently mentioned occasions to wash hands were before a meal (99.8%), after a meal (92.5%), after defecation (85.3%), and when hands looked dirty (75.6%). Among these four categories, before eating was most often spontaneously mentioned (proportion 86%). Hand washing after defecation was only spontaneously mentioned by 27% of the interviewees (Table 3).

**Table 3:** Knowledge, attitude, practice, and beliefs related to hygiene behavior, latrine possession, and open defecation mentioned by the respondents.

	Total reported %	Proportion spontaneous^a^	Mean prominence^b^
**Reasons to possess a latrine (** ***n = *** **89)**			
Safety	75.3	0.42	1.07
Clean environment	68.5	0.51	1.03
Comfort	67.4	0.53	1.03
Avoid diseases	67.4	0.27	0.85
Privacy	65.2	0.29	0.84
Visitors	70.8	0.10	0.78
Elderly people	51.7	0.26	0.65
Modern lifestyle	18.1	0.12	0.31
**Time point of hand washing (** ***n = *** **427)**			
Before a meal	99.8	0.86	1.85
After a meal	92.5	0.53	1.42
Always when dirty	75.6	0.64	1.24
After defecation	85.3	0.27	1.08
Before preparing a meal	50.8	0.31	0.67
Before nourishing a child	42.2	0.13	0.48
After cleaning a child	38.9	0.10	0.43
**Problems associated with open defecation (** ***n = *** **384)**			
Safety	85.2	0.65	1.40
Clean environment	71.1	0.28	0.91
Hygiene	63.5	0.36	0.86
No comfort	62.2	0.37	0.85
Privacy	58.3	0.25	0.73
Elderly people	56.8	0.11	0.63
Visitors	58.6	0.05	0.62
Drinking water	52.1	0.04	0.54
**Reason not to possess a latrine (** ***n = *** **334)**			
No technical comprehension	51.1	0.91	0.98
Traditional habit	24	0.70	0.41
Soil not stable	9.9	1.00	0.20
No material	12	0.60	0.19
Low priority	6.9	0.52	0.10
**Reason to practice open defecation (** ***n = *** **320)**			
No latrine in the household	88.8	0.95	1.73
Traditional habit	43.4	0.66	0.56

The study was carried out in the Taabo health demographic surveillance system in south-central Côte d’Ivoire in July 2011.

a Proportion of categories reported spontaneously.

b Mean prominence based on values assigned to each category (0 = not mentioned, 1 = probed, 2 = spontaneous).

Place and defecation frequency index assessed on a semi-quantitative scale, stratified by possession of a latrine, are summarized in Table 4. Study participants frequently reported to defecate into the nearby bush or open plantations. People living in households with latrines mostly used them, but they also practiced open defecation. Members of households without a toilet most of the time defecated in the open. A significant difference of defecation frequency could only be found between households possessing a latrine and households without a latrine for the nearby bush (0.73 *vs.* 3.28, *p*<0.001) and latrine (3.38 *vs.* 0.05, *p*<0.001), while no significant difference was found for the plantation (1.64 *vs.* 1.68, *p* = 0.969).

**Table 4:** Defecation behavior assessed with the parameters place and frequency, stratified by the abundance of household-owned latrines.

Place and defecation frequency index	Total households^a^	With latrine^a^	Without latrine^a^	P-value^b^
Near bush	2.75 (0.09)	0.73 (0.14)	3.28 (0.08)	<0.001
Plantation	1.67 (0.08)	1.64 (0.15)	1.68 (0.10)	0.969
Latrine	0.74 (0.07)	3.38 (0.12)	0.05 (0.02)	<0.001
Shared latrine	0.28 (0.04)	0.17 (0.08)	0.30 (0.05)	0.069
River/pond	0.06 (0.02)	0.03 (0.03)	0.07 (0.02)	0.207
Behind the house	0.05 (0.02)	0	0.07 (0.03)	0.173

The study was carried out among 431 households in the Taabo health demographic surveillance system in south-central Côte d’Ivoire in July 2011.

a Frequency of defecation (defecation frequency index) assessed on a semi-quantitative scale (0 = never, 1 = irregular, 2 = regular, 3 = often, 4 = always) for each place of defecation. Frequency is indicated as means (standard error in brackets).

b P-value assessed with Wilcoxon rank-sum test.

Most household members said that they need a latrine (98.5%) and nine out of 10 interviewees perceived open defecation as a problem. The most frequently stated reasons why a household does not have a latrine were the high cost (51.1%), traditional habit of open defecation (24.0%), not all of the required material locally available (12.0%), and soil not stable enough or the groundwater table too high for a durable construction (9.9%).

There was poor knowledge of schistosomiasis and parasitic worms in general (Table 5). Only 64.0% and 49.3% stated that prevention of schistosomiasis and parasitic worms, respectively, is possible. Open defecation was most frequently practiced because households simply did not have a latrine (88.8%), or because a deeply rooted tradition of open defecation (43.4%). Perceived problems with open defecation were safety issues with regard to different dangers lurking in the bush such as snakes (85.2%), pollution of the environment (71.1%), lack of hygiene (63.5%), lack of comfort (62.2%), and lack of privacy (58.3%). The only category with more than the half spontaneous answers of all reports was safety (65.0%). The top reasons to own a latrine were safety (75.3%), provide a decent place to defecate for visitors (70.8%), keep the environment clean (68.5%), preventing the spread of diseases (67.4%), enhanced comfort (67.4%), and higher level of privacy (65.2%).

**Table 5:** Knowledge of prevention of urogenital schistosomiasis and intestinal helminths.

Schistosomiasis		
**Can you prevent yourself from getting schistosomiasis?**		
**(** ***n = *** **253)**	Yes	64.0%
	Don’t know	32.0%
	No	4.0%
**If yes, how?** (multiple answers allowed)		
**(** ***n = *** **162)**	Do not bath	58.0%
	Do not drink dirty water	53.7%
	Do not defecate in the water	22.2%
	Do not eat overripe fruits	4.9%
	Do not eat washed fruits	1.2%
**Soil-transmitted helminthiasis**		
**Can you prevent yourself from getting parasitic worms?**		
**(** ***n = *** **424)**	Yes	49.3%
	Don’t know	45.3%
	No	5.4%
**If yes, how? (multiple answers allowed)**		
**(** ***n = *** **209)**	Do not eat overripe fruits	38.8%
	Wash hands before eating	38.3%
	Eat candies	34.0%
	Wash hands after defecation	33.5%
	Taking medication	27.8%
	Wash fruits	27.3%
	Do not eat meat	13.9%
	Wearing shoes	4.3%

The study was carried out among 431 households in the Taabo health demographic surveillance system in south-central Côte d’Ivoire in July 2011. Questionnaires were applied on a household level and the question only asked if the participant stated to know the disease.

### Association of Parasitic Infection with Hygiene and Defecation Behavior

All significant associations between a specific parasite infection and hygiene and defecation behavior and demographic factors are summarized in Table 6. For several different parasitic infections (*A. lumbricoides*, *E. coli*, *E. nana*, *I. mesnili*, and *C. bütschlii*) Muslims had lower odds of an infection than their counterparts with other religious beliefs. Besides demographic characteristics, place of defecation and hand washing behavior showed statistically significant associations with intestinal parasitic infections, including hookworm, *T. trichiura*, *E. hartmanni*, *E. nana*, and *B. hominis*.

**Table 6:** Significant associations between parasitic infections and household assets, hygiene, and defecation behavior.

Parasite	Association	Adjusted odds ratio (95% CI)	*P*-value^a^
*S. haematobium*	Christian	1.00	
	Muslim	7.18 (2.60–19.80)	<0.001
	Animist	2.10 (1.47–3.00)	<0.001
	Hand washing for personal hygiene	3.50 (1.68–7.28)	0.001
	Use of pond water for hand washing	3.76 (1.75–8.08)	0.001
	Hand washing time points spontaneously correct answered	2.61 (1.52–4.49)	<0.001
Soil–transmitted helminths			
Hookworm	Latrine	0.63 (0.40–1.00)	0.050
	Hand washing to prevent diseases	0.75 (0.58–0.98)	0.037
	Defecation in the bush	1.70 (1.07–2.69)	0.025
	Children defecating in latrine	0.53 (0.34–0.83)	0.006
	Children defecating in the bush	1.64 (1.06–2.54)	0.027
*A. lumbricoides*	Christian	1.00	
	Muslim	0.27 (0.09–0.87)	0.028
	Knowledge of parasitic worms	0.39 (0.20–0.78)	0.008
*T. trichiura*	Hand washing to prevent diseases	0.37 (0.16–0.86)	0.020
	Children defecating in latrine	0.50 (0.25–1.00)	0.048
Intestinal protozoa			
*E. hartmanni*	Farmer	0.54 (0.33–0.90)	0.019
	Drinking water from pump	0.52 (0.29–0.94)	0.031
	Defecation in latrine	0.27 (0.11–0.67)	0.005
*E. coli*	Christian	1.00	
	Muslim	0.75 (0.64–0.88)	<0.001
	Fisher	1.57 (1.30–1.91)	<0.001
*E. nana*	Christian	1.00	
	Muslim	0.81 (0.67–0.99)	0.039
	Latrine	0.80 (0.66–0.97)	0.027
*I. mesnili*	Christian	1.00	
	Muslim	0.58 (0.40–0.85)	0.004
	Animist	0.80 (0.65–1.00)	0.047
	Other religion	0.57 (0.44–0.75)	<0.001
*C. bütschlii*	Christian	1.00	
	Muslim	0.51 (0.29–0.91)	0.023
	Animist	1.53 (1.09–2.15)	0.014
*B. hominis*	Fisher	0.61 (0.37–0.98)	0.041
	Hand washing to prevent diseases	0.71 (0.51–0.97)	0.030

The study was carried out among 431 households in the Taabo health demographic surveillance system in south-central Côte d’Ivoire in July 2011. Logistic regression analysis was used with village level exchangeable random effects. Variables included as potential confounders were age groups (<5, 5–14, 15–24, 25–40, and >40 years), wealth quintiles and sex whenever age, sex, and socioeconomic status were significantly associated with a given parasitic infection.

No significant associations for *E. histolytica/E. dispar* and *G. intestinalis* with household assets, hygiene, and defecation behavior have been found after correction for potential confounders (sex, age group, or wealth quintile).

a *P*-value based on Wald test.

## Discussion

The global strategy for the control of helminthiases emphasizes preventive chemotherapy [4], [10], [11], [37]. The impact of this strategy on morbidity control is undeniable [38]. However, there is rapid re-infection after deworming, and hence the importance of improved sanitation is widely acknowledged in the literature dating back almost 100 years [13], [15], [16], [19]. Yet, compared to preventive chemotherapy, relatively little attention is paid on improving sanitation and clean water in contemporary helminthiases control programs [17], [18], [39]. In the present study we assessed the prevalence (and intensity) of helminths and intestinal protozoa infections and associated these findings with the local KAPB in nine purposely selected villages/hamlets of the Taabo HDSS in south-central Côte d’Ivoire, where annual preventive chemotherapy against helminth infections is administered to the entire population. The most prevalent helminth infection was hookworm (33.5%), followed by *S. haematobium* (7.0%). Other helminths were encountered only rarely.

The investigated parasitic infection prevalences and intensities were much lower than some 10 years ago; initial hookworm infections in the Taabo area in the late 1990s/early 2000s were high (34.4–54.0%), while initial prevalences for *A. lumbricoides* and *T. trichiura* infections were low; 0–1.3% and 3.3–7.5%, respectively [40]. The reduction of the highly prevalent infections can partly be explained by the interventions carried out within the Taabo HDSS as well as preceding research and control activities against schistosomiasis [24]–[26], [41]–[43]. Indeed, our continuous research-cum-action activities pertaining to helminthiases in selected localities in the study area might have had a positive influence by reducing the incidence through improved knowledge about these otherwise neglected disease in the population. In previous work on schistosomiasis in western Côte d'Ivoire we found that our research activities considerably improved knowledge in the community [22]. Furthermore, while *S. haematobium* and *S. mansoni* infections are a problem for only certain localities due to the focal distribution of the disease, it can be tackled comparably easy once these foci are identified. In contrast, hookworm infections are more homogeneously distributed throughout the Taabo HDSS and considerable in- and out-migration and the challenge to reach high coverage with preventive chemotherapy are important underlying issues. It should be noted that, despite continuous control efforts through annual deworming, re-infection with hookworm occurs rapidly. Hence, there is a need to continue preventive chemotherapy, coupled with additional control measures to prevent rapid re-infection [13], [44], [45].

Two limitations of our study are offered for discussion. First, although duplicate Kato-Katz thick smears were performed on single stool samples in order to increase sensitivity of the technique [46] it is conceivable that the reported helminth infection prevalences are an underestimation of the “true” situation in the study area. The issue of missing low infection intensities based on microscopic examination of single specimens has been discussed before [47], partially explained by considerable day-to-day variation of helminth egg output [48], [49]. Other new diagnostic tools such as the FLOTAC technique [50], molecular approaches (i.e., polymerase chain reaction (PCR) [51]), or the collection of samples over several days should be considered in future studies to increase sensitivity [52]. Second, the low prevalence of infections with *T. trichiura* and *A. lumbricoides* made it difficult to draw conclusive evidence about the direction and strength of association between these helminth species and risk factors.

Several intestinal parasite infections showed significant association with socioeconomic status, confirming observations from western Côte d’Ivoire of significant disparities of parasitic infection status among study participants [53]. Hookworm, *T. trichiura*, *E. histolytica/E. dispar*, *G. intestinalis*, and *I. bütschlii* were more prevalent among the poorer wealth quintiles. Surprisingly, *S. haematobium* was positively associated with higher socioeconomic status and Muslim showed a higher risk than people with other religious beliefs. However, these observations might be explained by the focal distribution of urogenital schistosomiasis; 97% of all *S. haematobium* cases were found in Sahoua, situated in close proximity to the Bandama River. In this village, the majority of inhabitants are Muslims. Moreover, the average socioeconomic status of Sahoua is considerably higher than other study village and hamlets.

A generally good hygiene behavior (e.g., not drinking dirty water, hand washing after defection) was recorded, which undoubtedly impacts on parasitic worms. Knowledge of schistosomiasis transmission (e.g., swimming and bathing in Lake Taabo) is widely known (58%), while wearing shoes to prevent hookworm infections was rarely mentioned (4.3%). This lack of knowledge about hookworm transmission might explain the relatively high prevalence of this helminth species despite several rounds of deworming.

Open defecation was commonly reported by the study participants. Indeed, the habit of open defecation is so deeply rooted that it was also reported (at least partially) among households having a latrine. As expected, we found a significant negative association between hookworm infection and the use of a latrine, confirming results from a systematic review and meta-analysis [19]. Literally all variables related with latrine availability or use were associated with a significantly lower odds of certain helminth infections (most importantly hookworm), but also some intestinal protozoa infections (e.g., *E. hartmanni* and *E. nana*) [54], [55].

Sanitation and hygiene behavior have proven to be substantial contributors to a sustainable control of soil-transmitted helminthiasis, schistosomiasis, diarrhea, and other fecal-orally transmitted diseases [56]. However, the promotion of sanitary solutions and the improvement of hygiene behavior are of a higher complexity than the regular administration of anthelmintic drugs to at-risk populations, as the former entail many locally rooted socio-cultural idiosyncrasies. For example, the possession of a latrine does not necessarily mean that it is being used [19], [57]. In the current study, however, the participants living in a household with a latrine reported its use, but we did not further verify these self-reports through direct observations. Open defecation is still commonly practiced among households possessing a latrine, particularly when people pursue agricultural activities, often several kilometers away from home. Importantly though, open defecation while pursuing agricultural activities was not associated with a higher odds of helminths and intestinal protozoa infections, which is in contrast to open defecation within a human settlement (village or hamlet) and in close proximity to open water sources. Population density in human settlements is higher than on plantations, and hence contaminated feces in the village/hamlet are a source of infection for villagers. Nevertheless, open defecation is not desirable in any case and should be stopped for the reason that plantations and agricultural fields in close proximity to open water bodies could contaminate the environment.

The main reasons advanced by interviewees regarding the possession of a latrine were issues of safety, privacy, enhanced comfort, clean environment, and hygiene. Indeed, households that attributed importance to these issues were more likely to have a latrine. Health-related reasons such as hygiene or prevention of disease were frequently reported, but only a small number of interviewees mentioned such reasons spontaneously, indicating that health-related issues were perceived as less important. Although, everyone stated the need for a latrine, not everyone was motivated to build one, mainly because the construction of latrines was perceived as an expensive undertaking. Furthermore, open defecation was seen as a traditional behavior that the whole village/hamlet is practicing. Overall, we found that health-related reasons played a minor role in the decision-making process. Therefore, health education interventions are necessary to increase the motivation of change or sanitation promotion focusing on these socio-cultural and socioeconomic reasoning and taking the whole spectrum of the villagers’ concerns into account [23], [33], [58].

Most people reported that they regularly wash their hands, but myriad reasons for hand washing were given. However, villagers seemed not to associate disease prevention with general cleanliness as the two elements were mentioned separately. Our analyses revealed that the most important factor for regular hand washing was the type of preceding (e.g., field work or defecation) or subsequent activity (e.g., food consumption). “Before a meal” was mentioned by almost all interviewees and indeed with a high proportion of spontaneous responses, which is important for the prevention of diarrheal diseases [59]. Although hand washing after defecation was reported by 85.3% of the interviewees, it was reported spontaneously only by a small proportion of study participants. This could indicate that people only answered yes to please the interviewer, but in reality, they do not wash their hands regularly after defecation. Failing to wash hands after defecation favors the transmission of fecal-orally transmitted diseases [60].

In conclusion, our results show that the use of latrines is associated with lower odds of hookworm infection. The study also indicates that morbidity due to soil-transmitted helminthiasis and schistosomiasis has been greatly reduced in the Taabo HDSS and preventive chemotherapy certainly played a key role [24], [43]. However, there is rapid re-infection after deworming, and hence integrated control approaches are necessary to keep the prevalence and intensity of infection – and thus morbidity – low. The parasitological and questionnaire data reported here will serve as a benchmark to monitor the effect of CLTS and hygiene education with the goal to reduce and interrupt the transmission of helminth and intestinal protozoa infections.
